# Supplementary material for: Intra- and inter-rater reliability of joint range of motion tests using tape measure, digital inclinometer and inertial motion capturing
Source: PLoS One. 2020 Dec 10;15(12):e0243646. doi: 10.1371/journal.pone.0243646 (PMC7728246; doi:10.1371/journal.pone.0243646)
Supplement: Attachment — Submitted filename: Reviewer 2.docx [file pone.0243646.s001.docx]

**Response to Reviewer 2:**

Thank you very much for taking your time to assess our work and your suggestion. Please find the response to your comments below. In general, we learned, that there has been a misunderstanding of the aim of the study. We did not intend to conduct a validity study, which we apparently failed to describe more precisely in the first version of this manuscript, possibly because we are non-native English speakers. In the revised version, we emphasized on rephrasing the entire manuscript more distinct. We aimed at conducting range of motion data of low cost devices and IMC in order to obtain intra- and interrater reliabilities for both methods in order to give advice for clinical applications.

**Reviewer #2: The presented study investigates the inter- and intra-rater reliability of three different techniques to assess RoM based on five standardized movement tests. The authors highlight that validity and reliability is key for adequate practical application of RoM assessments in a clinical environment. They therefore compare the results of two low cost techniques (digital inclinometer and tape measure) against a kinematic measurement system (inertial motion capture). Based on the results (ICC and measurement errors), the authors recommend both low cost systems for the use in medical assessments.

Major issues:

In its current form it is not clear enough stated what is the precise aim of the investigation. Is the IMC system assumed to be the “gold standard” to validate the use of the two low cost techniques? If so, the validity and reliability of the IMC system against the laboratory “gold standard” for movement assessment (until today, that is still a marker based optoelectrical 3D-camera system) needs to be stated and discussed much more thoroughly.
The experimental design is in general thoroughly described; however, some aspects will benefit of more details to fully understand all performed comparisons (see points below).
In terms of conducted statistical tests it is recommended to also incorporate Bland-Altman analyses (with corresponding plots). This would allow to assess systematic and random errors between the systems given in its actual measurement units. A more critical discussion in regards to the findings of this study is required.**

We did not include Bland Altmann plots, because we are aware that the low cost devices and the IMC system does not measure exactly the same construct. For example, in the tape measure tests we used the distances from the hand sensor, which is placed centrally on the back of the hand. In the lateral flexion we could calculate the distance to the floor because subjects were standing on even ground and we could use the single level scenario. In the finger-tip-to floor test we had to use the multi level scenario because subjects were standing on a little bench and not on even ground. In this scenario, a calculation to the floor is not possible. Therefore, we calculated the distance between the hand sensor and the foot sensor. It is also impossible to gain data of the fingertips in the IMC. Our aim was to best reproduce the construct used in the TM device, so the aimed range of motion is reflected likewise. Nevertheless, in the revised version of this manuscript we have included scatterplots which show the relationship between the methods (Fig2).

**Minor issues/comments:

- Line 75: if the IMC is your gold standard, then you will need to provide further details of its validity/reliability compared to highly standardized laboratory methods.**

Thank you for the suggestion. Although this is not a validity study we now included information on the comparison of the OMC and the IMC in the introduction section.

**Line 92: this figure only shows the graphical representation of the IMC software. It would be helpful to also provide pictures of an exemplary participant where the actual measurements (IMC vs DI or IMC vs TM) are performed**

Thank you for mentioning. We now included a revised version of Fig1, where the exact measurements on an exemplary subject are shown. The subject wears the measurement suit and a rater measures angles and distances.

**-Line 101: its recommended to always use the same amount of decimal points (And in this case even rounding up to the full year would be fine for this kind of data)**

Thanks for the hint. Done.

**Line 117 and following: here the reader needs more information about the system. Are there other resources about measurement precision than given by the manufacturer (independent scientific investigations). The provided statement is too general. Measurement precision depends on various calculations and will much likely depend on the site and the movement (as discussed by the authors in part later in the discussion).**

Thank you for mentioning, we have expanded on this in the introduction section.

**Line 128: What is meant by HD reprocessed? This needs to be introduced/explained**

We have added explanations.

**Line 137: Is there a source for that?**

Included in the method section.

**Line 152: This is not clear for the reader. This means 15 familiarization trials before 5 actual measurements trials? But later your spearman calculations are based on up to 550 number of repetitions.**

We have tried to clarify that section. *“In each test 25 repetitions were performed which were recorded simultaneously by the IMC system and the DI or IMC. The first 20 repetitions were recorded by the first rater but not included in any calculations as they were included as warm up in order to control for acute effects [32, 33]. For rater one, measurement 21 – 25 were included in the analysis [34]. Subsequently, the second rater measured another five repetitions. The order of the raters was randomly chosen.”*

Thanks for also mentioning the correlations. There was a mistake we know corrected. The new correlations were executed on only one repetition per subject. See the description of the new Table 4: *“The 21st repetition of rater 1 was used exemplarily; only measurements where synchronicity could be ensured were included.”*

**Line 168 and following: as mentioned above, an exemplary picture with the methods applied would much likely be appreciated by the readers. Same applies for the other tests.**

See new Fig 1.

**Line 174: what is meant by “length in 3D”?**

Now rephrased: *“The arm length (distance between humerus head and wrist)*…

**Line 223: How much did the data deviate from normal distribution. This also affects the outcome/validity of the ICC calculations.**

In the first version of the manuscript there was a mistake in the calculations of the correlations. Now we used only one repetition of each subject to exemplarily show the correlation. Here, all data was normally distributed. In the ICC calculations five measurements of each rater were included.

**Line 257 and following: The tables are sometimes difficult to read. It is recommended to separate the different statistical tests by thicker lines (or in a different way), e.g. between “Measurement error” and “95% Confidence interval”;**

Thanks for mentioning, we reformatted the tables.

**All tables: used abbreviations need to be explained in a legend for each table**

Done.

**Table 1: How are these high ”n’s” possible? That is not sufficiently explained**

Thank you for mentioning. This was a mistake, we initially used all data, including the 15 initial repetitions. Now we used only 1 repetition per subject. See new Table 4.

**Table 2: The unit of measurement for “Measurement Error” and “repeatability” is missing, or? Both outcomes are not well described within the manuscript (and only short in the given reference)**

We have included the units in the tables and tried to enhance the statistical analysis section.

**Table 4: the denotation “DI/TM” might be changed to “DI or TM”**

Done.

**Line 276: language: why is a comparison justified? This is not well expressed. Low Spearman’s rho results (especially for the Thomas test) need to be further discussed.**

Thank you for the hint. We have rewritten the whole section.

**Line 279 and following: but there are more potential methodological reasons for that.**

Thanks for mentioning. We have rephrased the entire discussion, calculated new correlations.

**Line 281 and following: Statement not clear**

The entire section has been rephrased now.

**Line 331: So in other words the comparison is potentially based on invalid data! That should be investigated and clearly stated**

The authors are not sure, what comparison you mean, since line 331 is empty. You potentially mean Line 313, which is about the Thomas test. This confusion was based on the wrongly calculated correlations. In the revised manuscript with the newly calculated correlations this does not occur anymore.

**Line 334 and following: measurement units?**

Included.

**Line 356: If these methods are adequate to measure individual training progress, then it would be good to discuss how precise exactly they are. What is the minimal detectable change? Statement regarding the identification of individual training progress might however be beyond the possibilities of this study.**

Thank you for the advice. We included data on the measurement precision in the rewritten discussion and were more careful considering the training progress evaluation.
