# Supplementary material for: Intra- and inter-rater reliability of joint range of motion tests using tape measure, digital inclinometer and inertial motion capturing
Source: PLoS One. 2020 Dec 10;15(12):e0243646. doi: 10.1371/journal.pone.0243646 (PMC7728246; doi:10.1371/journal.pone.0243646)
Supplement: Attachment — Submitted filename: Response to Reviewers.docx [file pone.0243646.s002.docx]

**Response to Reviewers**

#Second Review

Thanks again for you’re your time and effort in reviewing our manuscript. We address the minor revisions in the latest clean version of the manuscript. Therefore, the line numbering is different. I added the correct line numbering in the specific comments.

Reviewer 1:

Specific comments
line 121: please remove this p-value regarding the RMSE.

- Done. See line 97.

Line 305-306: it might be worthwhile to say in the sentence that the XSens software calculates the relevant joint angles for all parts of the body assessed in the study with the exception of the shoulder test.

- Done. See line 251-254.

Line 360 – 364: I am a little bit unsure what is meant by the comparison of the measurement systems if that is not a validity question. Does this still reflect the revised title and aim of the study and my initial concerns regarding a validity comparison? Or are you using such a comparison to make it clear that these different measuring systems are not directly comparable and shouldn’t be interchanged?

- Sorry, that term “comparison” must have been missed in the first revision process. Of course we agree that it is not a validity study. We rather wanted to describe the relationship between the systems by showing the Pearson correlation. In the results section we already changed the wording in the first revision. Now we also changed it in this section, see line 287-289.

Table 1 – 2: in regards to the measurement error, repeatability and COR scores, you have listed the units as (cm/o). This suggested the reader that you have some new unit of measurement that combines both distance and angles for all of your tests. However, I feel what you’re trying to represent here is that some of these measures are distances and others are joint angles. Therefore, can you make it clearer in these tables which unit of measurement reflects each of the different tests?

- Thanks for mentioning, we have already described that in the description of the tables, but we agree it should be visible in the table itself. We changed that in the tables.
